# Sport can do better: female athletes' perspectives on managing menstrual and hormonal contraceptive cycle-related symptoms

**DOI:** 10.3389/fspor.2025.1597469

**Published:** 2025-05-26

**Authors:** Sara Chica-Latorre, Catherine Knight-Agarwal, Andrew McKune, Michelle Minehan

**Affiliations:** ^1^ Research Institute for Sport and Exercise, University of Canberra, Canberra, ACT, Australia; ^2^ Department of Nutrition and Dietetics, Faculty of Health, University of Canberra, Canberra, ACT, Australia; ^3^ Functional Foods and Nutrition Research (FFNR) Laboratory, University of Canberra, Canberra, ACT, Australia; ^4^ Discipline of Biokinetics, Exercise and Leisure Sciences, School of Health Sciences, University of KwaZulu-Natal, Durban, KwaZulu-Natal, South Africa

**Keywords:** female athlete, sportswomen, menstrual cycle, symptoms, qualitative, performance, perceptions, symptoms management

## Abstract

**Introduction:**

Menstrual cycle-related symptoms (MCS) affect female athletes' wellbeing, quality of life, sports participation, and performance. Hormonal contraceptives (HCs) moderate hormone activity, but do not necessarily alleviate MCS. Recent research indicates no significant effect of the MC or HC cycle on measurable performance, yet perceived effects on performance and wellbeing remain noticeable. Thus, this study aimed to understand how female athletes are impacted by and manage MCS, and how they want these symptoms addressed in sporting contexts.

**Methods:**

Using a constructivist paradigm approach, experiences and perspectives of 30 female athletes [aged 19–32, 18 naturally cycling (NC) and 12 using HCs] from 17 sports were gathered through semi-structured interviews and analysed using reflexive thematic analysis.

**Results:**

Four main themes were identified: (1) significant impacts of MCS on athletes, (2) perceived functional difference when MCS are present, (3) uncertainty about the effectiveness of management strategies despite trying multiple options, and (4) a desire for a supportive sport environment with meaningful discussions about MCS, though there is reticence.

**Discussion:**

These findings highlight the need to view MCS as more than a minor inconvenience, consider athletes' perceptions, and pursue more research on evidence-based management options and MC culture change in sports. Regardless of the sport, it is crucial to advocate for athlete-centred training loads and schedules that can improve the overall experience of athletes enduring MCS, helping them continue to participate in sport and perform at their best.

## 1 Introduction

The menstrual cycle (MC) is a fundamental aspect of female physiology characterised by fluctuations in female sex hormones (1). A variety of menstrual cycle-related symptoms (MCS), such as painful menstruation (dysmenorrhea), abdominal discomfort, low back pain, fatigue, headaches, and mood changes are known to affect females to different extents (2). Hormonal contraceptives (HCs) moderate hormone activity in order to prevent pregnancy and could offer therapeutic benefits (3), but do not necessarily mitigate or eliminate MCS (4). Thus MCS are also experienced in a HC cycle as side effects. Cross-sectional research suggests that approximately 80% of active women experience at least one recurrent symptom related to their MC or HC cycle, and these symptoms have potential to affect wellbeing, quality of life, sports participation and performance (4–7).

A call to action by researchers such as Bruinvels et al. (8) has seen a surge in research focused on the MC. Furthermore, understanding of menstruation biology, the MC and HCs within in the sports science community, is rapidly improving. Currently, the broad consensus is that there are no measurable differences in sports performance at different phases of the MC or in those using oral contraceptives [reviewed by (9, 10)]. However, this research has methodological limitations (9, 10), failing to capture the diverse and individual experiences of female athletes. While sports scientists question the value of tracking the MC and HC cycle, and whether or not to plan training around phases of the MC, there is growing recognition that the complex interactions between hormonal fluctuations, health, sociocultural beliefs, and athletic performance demand a deeper understanding from the perspective of female athletes (11).

Since 2020, a small number of qualitative studies have explored the experiences and perceptions of sportswomen regarding MCS [see for example (12)]. Many of the earlier papers were limited by quality issues but more recent papers have vastly improved in their execution of qualitative methodology (13). Although many experimental investigations have concluded that the MC does not measurably affect performance, others which have employed both quantitative and qualitative methodologies report that MC and HC use does affect women's capacity to train and perform (5, 11). Particularly, qualitative investigations have identified that athletes feel internal and external pressures to conceal and adapt to the challenges imposed by MCS in order to be able to continue to train and compete. Furthermore, stereotypes and stigma around the MC drives reluctance to communicate menstrual-related issues within female athletes' support networks, yet there is a desire to change conversations around the MC and HC use (13–15).

Recent qualitative investigations have highlighted the impact of MCS within a sporting context (12–15). However, these investigations have also raised unanswered questions about how female athletes cope with and overcome MC issues, and their perspectives on the best avenues for support. Exploring the experiences of female athletes from various cultural and sports backgrounds is crucial for capturing multiple realities and constructing transferable understanding of the MCS phenomenon (16), ultimately helping to generate ideas and options for better support and management. Therefore, this qualitative investigation aimed to understand how a diverse group of female athletes, from different sports backgrounds, are impacted by and manage MCS, and how they want MCS to be addressed in sporting contexts.

## 2 Materials and methods

### 2.1 Philosophical assumption

We conducted a qualitative study influenced by a constructivist paradigm approach that posits that reality is established through engagement with the social world, resulting in the existence of multiple realities (17). This aligns with our aim to develop a deeper understanding of the diverse ways that female athletes experience, perceive and attribute meaning to their MCS in a sporting context. We recognise that through the research process, knowledge is co-constructed between researcher and participant (18). We opted for reflexive thematic analysis as the most appropriate method to generate shared meaning between the participants' experiences and the researcher's analysis (19).

#### 2.1.1 The researchers

SCL (Colombian origin) is an early-career female sports dietitian, personal trainer, competitive active female, and doctoral student. This is her first qualitative project. CKA (Australian origin) is a female dietitian with abundant clinical experience in women's health but minimal experience working in sports environments. She is an experienced mixed method researcher. AM (South African origin) is a male strength and conditioning coach and exercise physiologist with over 25 years working in elite sport and research environments. MM (Australian origin) is a female sports dietitian with over 25 years working with elite athletes in high performance environments. She is an experienced mixed method researcher. The female researchers consider themselves to be minimally affected, moderately affected and severely affected by MCS. The diverse perspectives within the research team enabled a deeper interrogation of interpretations, with minor tensions arising between researchers with and without sport experience. The primary investigator's (SCL) positionality as an early-career sports dietitian and competitive female athlete may have fostered participant openness or, conversely, constrained disclosures due to inherent power dynamics. These potential influences were critically addressed through reflexive journaling and team discussions.

#### 2.1.2 Participants and recruitment

Purposive sampling was used to recruit female athletes, 18–35 years old, who at the time of recruitment participated in sports competitions and self-identified as healthy, and with regular MCs or HC withdrawal bleeds (frequent and between 21 and 35 days long). The study was advertised through sports institutions and club networks in Canberra and New South Wales (Australia) and via social media channels to reach a broad range of athletes. Female athletes were eligible for recruitment if they experienced at least one negative MCS for at least the past six MCs or HC cycles. Once identified as eligible, written informed consent was gained from participants prior to study enrolment. None of the participants had any prior relationship with the research team. The participant information explained that the project was part of a doctoral investigation to understand the impact of MCS on female athletes. Ethics was approved by the University of Canberra Human Research Ethics Committee (13381).

### 2.2 Data collection

Semi-structured interviews were used to explore the experiences and perceptions of female athletes regarding MCS. An interview guide was developed based on literature (12) and discussion with the research team. The guide (Supplementary 1) consisted of three-parts (1) introductory questions: demographic and sports-related; (2) main questions: MC/HC status, MCS, MCS management strategies; (3) menstrual literacy knowledge acquisition, perception on ease of menstrual-related communication, and general questions from participants. SCL completed two preliminary interviews before formal data collection commenced for interview guide testing and to develop interview skills capable of capturing rich data. MM and CKA reviewed the interviews and provided feedback on interview technique. The phrasing of some questions was refined during this process to ensure questions were understood and prompted insightful responses. The primary investigator (SCL) conducted all semi-structured interviews using the virtual communication platform Microsoft Teams (version 1.6.00.4472). Throughout interviews, the majority of questions were kept deliberately open, providing cues for participants to talk with a minimum of interruption and without judgement. Participants were advised that they could decline to answer any question/s and all information related to MCS was relevant. Interviews were conducted between August 2023 and March 2024, and were transcribed automatically by Microsoft and manually cleaned for accuracy by SCL. SCL and MM reviewed transcripts after approximately every 5 interviews to ensure that quality data was being collected. Recruitment continued until SCL and MM agreed that sufficient quality data had been collected to explore the phenomenon of MCS in female athletes. Malterud and colleague's (20) model of information power was considered when making judgements about when to cease interviewing.

### 2.3 Data analysis

A reflexive thematic analysis recognises the contribution of the researchers' subjectivity as a critical resource for theory engagement and data interpretation (19). It also facilitates theoretical flexibility, allowing us to meet the aims of this study. SCL, CKA and MM independently worked through a process of data familiarisation, inductive coding and generation of initial themes. We conducted a group white-boarding session to interrogate theme generation. We initially categorised data into topics then stepped away from the analysis for a period to allow for meaningful consideration of themes across the data. A series of discussions over a 3-month period strengthened the analysis process. SCL kept a comprehensive audit of data that contributed to each theme via an Excel spreadsheet. We further refined the naming and articulation of themes through the writing process. AM reviewed and interrogated the final findings. Participants were not involved in the analysis process.

### 2.4 Quality

To optimise the trustworthiness of our research we considered Braun and Clark's guidance on quality reflexive thematic analysis (21). The research team regularly challenged ourselves to ensure we were collecting rich, quality data and conducting authentic reflexive thematic analysis. We particularly interrogated whether we were generating thematic findings rather than merely categorising data. Our research is also reported in consideration of Tong and colleague's consolidated criteria for reporting qualitative research (COREQ) (2007) (22) (Supplementary 2). However, we recognise that some components (e.g., approaches to saturation and data coding) of COREQ do not fully align with the philosophical underpinnings of reflexive thematic analysis.

## 3 Results

A total of 30 healthy sportswomen from 17 sports were interviewed. Participants were invited to review the transcript of their interview prior to analysis however none completed this review. All participants agreed to have their data considered in the final analysis. The McKay et al. (23) participant classification framework was used to classify the level of athletes. Participant characteristics are summarised in Table 1. Interviews ran between 45 and 60 min. Reflexive thematic analysis generated 4 main themes with a total of 9 subthemes summarised in Table 2. Overall, the results indicate that female athletes feel functionally different and substantially impacted by MCS. Athletes have tried multiple strategies to manage their MCS yet feel uncertain about the effectiveness of options. Participants envisage a sport environment with better understanding of the way female athletes are affected by MCS and a culture of meaningful conversations about support options.

**Table 1:** Pooled participants' demographic details.

Age (years)	Sport, number of participants per tiers (Tier 2: trained/developmental; tier 3: highly trained/national level; tier 4: elite/international level) (23)	Naturally cycling (NC) OR hormonal contraceptive (HC) use	Cycle tracking (NO/YES) and methods used	Record menstrual cycle-related symptoms (NO/YES)
Mean = 25.9	Triathlon (Tier 4, *n* = 3; Tier 2, *n* = 2);	NC, *n* = 18	NO, *n* = 9	NO, *n* = 15
Median = 26	Ultimate frisbee (Tier 2, *n* = 5);	HC users, *n* = 12	YES, *n* = 21	YES, *n* = 15
Range = 19–32	Water polo (Tier 4, *n* = 3);			
	Soccer (Tier 3, *n* = 1; Tier 2, *n* = 2); Powerlifting (Tier 4, *n* = 1; Tier 3, *n* = 1); Olympic weightlifting (Tier 2, *n* = 1); Running (Tier 2, *n* = 1); Pole sport (Tier 3, *n* = 1); Canoe polo (Tier 3, *n* = 1); Irish dancing (Tier 3, *n* = 1); Bodybuilding (Tier 2, *n* = 1); Rugby (Tier 2, *n* = 1); Squash (Tier 4, *n* = 1); Sailing (Tier 4, *n* = 1); Hockey (Tier 3, *n* = 1); Figure skating (Tier 3, *n* = 1) Netball (Tier 2; *n* = 1)	Intrauterine device (IUD) Mirena ®, *n* = 6 Oral contraceptive pill (OCP): Femme-Tab ED 20/100, *n* = 2 Oral contraceptive pill (OCP): ELEANOR 150/30 ED, *n* = 1 Oral contraceptive pill (OCP): Yasmin ®, *n* = 1 Oral contraceptive pill (OCP): LEVLEN® ED, *n* = 1 (OCP): Estelle 35 ED, *n* = 1	Apple Heath, *n* = 7 Ōura ring, *n* = 2 Calendar based, *n* = 2 Garmin connect, *n* = 1 Basal body temperature (BBT), *n* = 2 FitRWoman, *n* = 2 Clue app, *n* = 2 Flow app, *n* = 3 Training peaks, *n* = 1	

**Table 2:** Summary of themes and subthemes with quote illustrations describing the way female athletes feel about menstrual cycle-related symptoms (MCS).

Theme	Subthemes	Quote Examples
Experience of MCS REAL TALK: Unveiling the realities of symptoms	MCS descriptions	“I get really tired, almost feel hungover. But it's like I'm not physically tired. It's just like mentally, I'm tired and just feel like cloudy.” (P2)
	Attitudes and concerns	“It's annoying because you feel that you need to be at a certain point in your performance, in your hype and you know everything. So, you sort of expect something from yourself, which maybe you're not giving.” (P21)
Effects on perceived sports performance GAME ON GAME OFF: My body feels like it is performing differently	Missing/modifying (or not) training/comps	“I was determined not to let my period symptoms affect training. If that makes any sense… because I guess we've been taught or like my mindset is that it shouldn't affect our training. But there are parts of it [symptoms] that do [affect training] and how to manage that better? and not feel like you're slacking off from training.” (P12)
	Negative perceived quality of training	“I feel like I still try and put everything I can into it [training], but I don't feel like I'm getting the return back if that makes sense… I'm trying my best knowing that I'm just not at my best and then so things just don't kind of, just the timing is off and you're trying your hardest, but you're just slow, even though you're trying to be as fast as you can.” (P12)
MCS management strategies SYMPTOM MANAGEMENT: Uncertainty about effectiveness of options	Applied strategies	“Previously I would take (non-opioid analgesic) or (non-steroidal anti-inflammatory) to deal with my cramps and back pain… (non-opioid analgesic) doesn't work. (non-steroidal anti-inflammatory) lasts very little time… I would rather just deal with it.” (P25)
	Monitoring	“Knowing the cycle helps me define why I'm feeling the way I'm feeling and what's going to happen.” (P10)
	Perspectives on HC use	“I decided to get what was recommended [the IUD] because actually I started on the pill and I didn't have a great experience with that*…* just because I could just tell, it made me feel not myself, like I just didn't feel like the nicest version of myself. Like just made me feel a bit irrational*…* I just felt a bit crazy, which wasn't usual for me to feel like how I was feeling, and it was when I went on the pill.” (P12)
Sociocultural aspects SPORT CAN DO BETTER: Desire but reticence to advance communication and support in sport	Communication	“As an athlete, depending upon the background of the athlete, there can often be a stigma about talking about your period… the coach should ideally talk about that and sort of break down that stigma.” (P8)
	Desire on progress	“It's really nice when the male coaches and people involved in your sport are trying to learn more so they know how they can help you. But there is a big difference between learning and actually applying things.” (P12)

### 3.1 Real talk: unveiling the realities of symptoms

The way female athletes experienced MCS differed from athlete to athlete and within each athlete from cycle to cycle. However, collectively participants described MCS as a collection of interconnected physical and psychological symptoms that had a profound impact. The breadth of symptoms and the way they are experienced by female athletes is summarised in detail in Supplementary 3. MCS were described by athletes who were naturally cycling as well as those using HCs. Noteworthy is the intense pain experienced by participants. For example, abdominal cramps that feel like “*two closed fists*' or cramps that are '*so bad, I have to throw up*'. Other discomfort such as breast tenderness that is '*so painful I dread it*' and joint pain that makes it “*difficult to move*” was also described. Participants described psychological symptoms (summarised in Supplementary 3) including ’*social withdrawal*', “*negative self-hatred*”, “*feeling downtrodden*” and “*finding decisions harder than I normally would*”. One participant reported that MCS led to feeling *“like I have a lower emotional resilience, if that makes sense. So, something at another time in my cycle that wouldn't bother me or make me upset, would bother me and make me upset*” (P2). Collectively, physical and psychological MCS had a major impact on overall energy levels with one participant explaining that it '*sucks the life out of me*'. The impact of symptoms compounded to impact other aspects of wellbeing. For instance, cramping compromised sleep quality with one participant exclaiming “*Sometimes I feel ohh! my concentration's not great because I'm tired because I didn’t have a good sleep last night because of my cramps”* (P23). Participants explained that the severity of symptoms tended to vary from month to month, as one participant outlined “*I think that they [symptoms] vary. It's sort of like a scale between not getting them at all, getting them at a moderate intensity, and then at a higher intensity… Stomach cramps are the most variable; sometimes little to none at all, and other times it's just like there's a war going on in my uterus*” (P8).

### 3.2 Game on game off: my body feels like it is performing differently

Participants perceived their training and competition performance to be affected by MCS. Physically, functionally and mentally they feel different.

This is supported by the following participant who claimed “*Everything's more tight and I think therefore slower. You keep your legs in a different range and you just walk differently and you can’t run that good. Not use your body the way you normally use, like stretching out or reaching for something. It's just different, not smooth*” (P18). Likewise, a powerlifter explained “*When I'm premenstrual, I usually feel unwell because I'm bloated and everything. I usually feel like I can’t brace as well, like my performance might not be the same or good as expected, but I don’t feel like I'm executing the lift the same in the sense that it's a bit more difficult for me to feel that same bracing tension*” (P21). Painful symptoms made some aspects of training and performance more uncomfortable, “*When I was riding a bike it's very difficult because I am in a position that you're already pressing on that area of the abdomen [lower area] … so I felt very unwell…*” (P7). Heightened pain sensitivity affected women in contact and tackling sports, such as rugby, “*Blows [tackles] even hurt more, it makes you too heavy, too heavy to play*” (P26). Participants also described physical differences in temperature regulation when bleeding. For example, one participant summarised “*I did the same race two years in a row… the second year, even though I was definitely a lot fitter than I had been the year before, ran slower because I basically just overheated in the race*” (P8). As a result, the physical impact of MCS caused frustration as P11 summarised “*cause you feel like why is this not going to plan? I might have my sleep in order. I might have my eating in order. I've like prepped, you know, pretty consistently to get to where I need to be in training, but then there's this underlying variable that I can't control like…* *just impacts things and it is sort of frustrating cause I just want it to move how it should be moving considering I put in the effort for it to move like it should be moving*”.

In addition to physical function, participants reported perceived differences in psychological and mental function when affected by MCS. They described how lower motivation, increased self-criticism and reduced focus added a “*mental battle”*' (P5) and “*mental load*” (P28), which imposed additional challenges to performance during training and competition. Participants talked about how “*being stuck in their own head*”, affected reaction time and decision-making “*It's like we always have in the sport the attitude of, you know, if you make a mistake, fine. But then you need to go and fix it like you need to bounce back and go and chase the ball or do whatever it needs to do. But if you’re going, oh, why did I do that? You know I can’t do anything. Then you’re not focusing on that next step and like getting back from it, you’re just getting stuck in your head*” (P16). Similarly, some participants reported that “…*if you're not really focusing and concentrating on what you're doing, then you kind of like just create more opportunities to hurt yourself. Like, if you're not really thinking about where you're putting your feet and things like that, then there's just like more risk, I guess that you might, you know, roll your ankle or something like that*” (P24). Additional mental distraction was experienced by having to manage the practicalities of bleeding as P28 summarised “… *obviously even logistically training when it's really heavy the first two days if I'm out training for six hours like just having a change of sanitary stuff is like so annoying*”. Likewise, another participant commented, “*In training I take longer because I keep going to the toilet… I don't know if I'm just like overflowed ‘I think I should go to the bathroom’. So you're checking a thousand times, and obviously it distracts*” (P21).

Pragmatically, participants recognised that they had to compete regardless of MCS, “*I know that you're not going to feel your best at every single game. That is just impossible… So I do feel frustrated [with symptoms], but I also kind of just categorise it into, well, it's part of being an athlete and it's the reality of being a female athlete”* (P2). However, competing with MCS was universally considered sub-optimal. Participants described being able to perform at a high level while experiencing MCS, however, still questioned if their performance was compromised. This is illustrated by P30's recollection of a competition day “*I had my period during a competition. I'll say it was awful because I had the added-on stress about me just bleeding through all my tampons… I feel like I didn't need it on top of already questioning a lot of my abilities on contest… I did come first in it, but I remember when we got the scores back, I felt like I didn't do as well as I could…*”.

Mostly, there was reluctance to modify training conditions during symptomatic times. Getting through training while struggling with MCS was seen as part of being an athlete and important for being able to cope with different competition scenarios, “*Who knows if I'd get cramps during a competition or match day? So I was just trying to treat it like that… So I did see the benefit of that, but it was, yeah, very distracting*” (P12). Others expressed acceptance about poorer quality performance, rationalising that they would compensate when feeling better in future training, “*… oh I didn't run as far today, so I need to make up for those missed kilometres like next time… so I think there is a flow on effect and kind of like putting a little bit more pressure on the following sessions to be better and to be at a higher quality*” (P23). Athletes recognised that they were more likely to function better once their symptoms eased, “*Suddenly, like, not feeling affected by menstruation. My energy is up, I'm sleeping better, I'm in a better mood just generally… While the training starts to feel better. So that's obviously a positive feeling*” (P21). This was echoed by P5 who explained, “*Like after the pain on my period, after the first, let's say two days… I feel really good in training and if I test or race then I feel really confident because I know it's gonna feel good, but also, I can get the best out of me from a field point of view and it feels easier to do it…* *I will say that mental aspect of knowing that I'm healthy”* (P5). This knowledge of the way their body functioned, gave some participants that confidence to modify their training around their MCS, “*I know that there's this period where like I have to make adjustments because I'm just not gonna be feeling 100% myself… it's like you have to rest at some point and rest is obviously a very important part of training and performance…* ” (P3). Some athletes had developed comfort with modifying training as they matured “*When I was a bit younger, earlier 20s, I would be more willing to just kind of ignore that and throw myself at it and almost leave myself to burn out at the end of each tournament. Whereas now I'm more happy to train within what I know my body is letting me do and what it doesn't wanna do*” (P27). Other athletes, particularly in team sports, worried that having to modify training would cause them to not be “*good enough”*, or to “*fall behind”*. They questioned whether it was legitimate to miss or modify training if “*like I'm not sick”* (P7). P13 describes this conflict, “*there was that dichotomy between knowing that rest is good, like recovery is important and good, to then being like, oh, but when I go back to training, am I going to lose my gains, or would I just not be able to run quite that far, or push quite that hard for a little while*” (P13).

### 3.3 Symptom management: uncertainty about effectiveness of options

Participants utilised a variety of strategies for managing MCS, which broadly included pain relief (pharmacological and non-pharmacological), dietary modification, self-help strategies (i.e., meditation, self-empathy), cycle tracking and use of HCs. Nevertheless, there was uncertainty about the effectiveness of management strategies.

Managing pain associated with MCS was a key priority with the following participant claiming: “*I always prefer to drink hot water or put something hot on my abdomen if possible. But because of the immediacy, like when I can't stay in the house, even if I don't want to do that, I'm going to take pills [painkillers] to make it better fast*” (P7). Many participants expressed frustration with the ineffectiveness of pharmacological pain management, “*Previously I would take (non-opioid analgesic) or (non-steroidal anti-inflammatory) to deal with my cramps and back pain… (non-opioid analgesic) doesn’t work. (non-steroidal anti-inflammatory) lasts very little time… I would rather just deal with it*” (P25). For others, despite drug therapy being effective it was utilised sparingly with the following participant claiming “*Painkillers, I only take them if I really really have to. I try to avoid it, so it might happen like maybe once a year or twice, but no more than that…* *I have this aversion against painkillers also when I have a headache in other contexts. It's more emotional than a rational choice, though*”. Overall, participants expressed a strong desire to find relief from pain but mostly felt the use of medication was not a palatable or effective option.

Participants widely recognised that their appetite and food preferences changed at different phases of their MC or HC cycle. Broadly, there was a desire to continue to consume a high-quality diet with emphasis on “*healthy*” options such as fruits, vegetables and wholegrains across the cycle, and uncertainty was expressed about whether “*giving in’* to cravings was beneficial or harmful. One participant noted “*Interestingly, this month I changed my diet a little bit…* *and weirdly, no cramps beforehand, not as much breast tenderness beforehand, I didn't binge on chocolate this time. Um and, I just felt like it hit me less than what it normally does*” (P1). Some participants reported actively modifying the amount or type of nutrients they consumed to prevent MCS. For example, additional iron (through food or supplements) was considered useful to combat fatigue “*I take iron supplements and as well and I make sure I have enough protein… I feel like the symptoms that you usually get around your period and feeling sluggish and crappy, it could be like from that [not having enough iron and protein] because I know that I purposely try to do those things so it doesn’t hinder my training”* (P22). Magnesium (supplement) was used to help with disturbed sleep, “*…* *I'm like having like magnesium powder before bed. Sometimes if I know I've got a big competition, especially if there's travel involved. So, you’ve gotta disrupted sleep. So just having something like that to try and at least even if it's placebo*” (P16). Increased hydration was also considered to help improve the sensation of menstrual cramps and moodiness, reduce skin blemishes, and (for some) combat overheating, as two participants explained, *“I just try to drink as much water as possible… I think when I'm on my period, I think it's almost like I'm feeling tired and, you know, stiff and sore and grumpy and maybe that's because I'm, like, dehydrated as well like from like menstruating…”* (P24) and “*I sweat a lot more [in the days prior to bleeding] and so I'm working with my nutritionist on increasing my fluid intake just to sort of manage the results of that overheating and sweating a lot more and getting dehydrated a lot more.”* (P8)*.* Participants also spoke about strategically modifying their diet to work around MCS, “*I just don't feel as much like I have an appetite. I have to like make myself eat food a lot more. So, like on those days, [during bleeding] I probably like, will try and have a bit more during my session. So have it in the form of like a carbohydrate drink and stuff like that, so I don't have to try and eat as much outside of my sessions*”. (P28). Others described avoiding foods perceived to trigger adverse symptoms, “*If I'm feeling really like gross and crampy… I just know that I don’t want fatty foods… I almost feel like it's gonna make me feel worse*” (P24). Overall, dietary modifications were seen as important to manage MCS, however participants wrestled with best options.

Some participants viewed monitoring and tracking the MC and HC cycle as useful when preparing for the onset of MCS, “*Like I just got into the routine of knowing [her cycle] … So having that routine just took a bit of the mental thinking away from it so that you could kind of focus on just getting in the right spot for the game”* (P16). However, there was also recognition that tracking could cause additional stress when symptoms and bleeding deviated from tracking history. Others placed less value on tracking, conveying that it just created an additional mental load without any tangible benefit. Regardless of any insights gained from tracking, athletes had to *“soldier on”*, as expressed by P21 “*During training, I don’t really have a strategy to manage it [pain]. So, I sort of just brace and try…* *I basically just get annoyed and in a bad mood because I feel sluggish because the pain is horrible. It's not nice pain. But I'm training with pain.”*

Participants reported that HCs could be a useful option for managing some MCS, “*My experience with the Mirena [IUD] has been fairly positive. It reduces my period length. The pain that I get is also reduced slightly while it is still quite severe. It is much more bearable”* (P25)*.* Many other athletes described negative experiences with HCs, “*I've tried others [OCPs] and it's chaos… Either there is no period, or I get very upset, or I go crazy.*” (P10). Participants described testing to see if HCs were helpful “*It's been a journey with it [HCs] and you feel like you kind of just winging it”* (P12), and “*…* *it is a bit tricky with contraception that you don’t know if it's gonna suit you until you actually try it*” (P16). There was also uncertainty about whether HCs provided meaningful improvement or not, “*Every now and then people tell me, ‘I've stopped using it and I feel better’… so sometimes I'm wondering how that would be for me, but I haven’t tried it out yet*” (P9).

### 3.4 Sport can do better: desire but reticence to advance communication and support in sports

Participants voiced a desire for all involved in sporting organisations (but particularly coaches) to better understand how the MC and HCs affect athletes and for creating a culture of meaningful, non-judgemental communication, but reticence about engaging in conversations about their own MC or HC use was common.

Participants recognised the MC as a natural process and expressed not wanting to feel embarrassed or apologetic for it “*It's funny that when you start bleeding, as an athlete you try to do what you can to stop bleeding, like you’re just trying to stop your body from doing what it's actually supposed to do”* (P12). Participants identified that being open about menstrual health can make it easier to manage issues that affect training and performance as one participant summarised “*You're never gonna perform if you can't be healthy and then don't rush it. Like, take your time and do what you need to do, and that includes period management. That includes all of it and just be a little kinder because you'll get there if you just do it properly”* (P5). Participants recommended better, early education about the MC and HC use “*Many people struggle. When I was in school, the only thing I knew about other people was that they had cramps and took painkillers for it. I would have liked to know about the altogether symptoms and how they affect you”* (P18). They also wanted those around them (e.g., coaches) to be better informed, “*Coaches do need to know more about menstrual symptoms… just like an awareness that it can impact people differently… Letting people do what they think is gonna work for them… rather than just following one piece of advice”* (P24).

Within this athlete cohort, despite a high-level desire for improved consideration of MCS, participants typically acknowledged they avoid sharing menstrual-related information due to stigma and the uncomfortable nature of such discussions. They recalled coaches making inappropriate comments (e.g., describing menstruation as having “*a lady day*”) or assumptions (e.g., associating bleeding with weakness), leading them to feel less inclined to want to talk about their MC and HC use. One participant explained “*Like I have experienced coaches who have actually weaponised somebody's menstrual cycle in a gaslighting way… I've had a couple of comments where it was like, ‘Oh, you’re being really anxious at your time of the month or something. Is that your time of the month?’ So that sort of very stereotypical, like, ‘Oh, you're moody and you're expressing an emotion, so therefore you must be on your period”* (P13). Others considered it unnecessary or irrelevant to share menstrual-related information unless “*If it's gonna affect training or performance then I would have that conversation. But I think if it's not, then I kind of probably make that self-assessment of, OK, well it's not impacting then there's probably no need to have that conversation”* (P20)*.* There was also hesitancy about how much information to share, as P24 explained “*I would feel comfortable enough to be like ohh I'm on my period, but I don't think I'd be comfortable to go into any more detail than that… I don't want to come off as like weak or incompetent.”* In contrast, one participant explained that meaningful communication about MCS had been beneficial, “*My coach and I base my training kind of on my cycle. So, we'll do kind of a down period, like a recovery type week when I have the worst like PMS [premenstrual syndrome] type symptoms…* *it has been really helpful*” (P3).

Participants primarily called for coaches to take responsibility for changing discomfort about talking about the MC and HC use, “*As an athlete, depending upon the background of the athlete, there can often be a stigma about talking about your period… the coach should ideally talk about that and sort of break down that stigma”* (P8). Some female athletes perceived gender to play a significant role in how comfortable individuals are about discussing their MC and HC cycles and specifically identified “*older men*' as not understanding as summarised by one participant “*I've had predominantly female coaches for the past bit and so I'm comfortable going up to them and saying like I'm on my period right now…* *This upcoming season there's talk of us having a head male coach, don't know how that will go”* (P27). Participants challenged male coaches, in particular, to ensure they understand how the MC and HCs affect individual athletes and to create a culture for meaningful and respectful discussions, “*Female identifying coaches have a better handle on it because a lot of them are people who menstruate as well…* *Male identifying coaches who step into lead female identifying players need to potentially set that precedent and say we acknowledge that there will be athletes among you who are people who menstruate”* (P27).

## 4 Discussion

**Figure 1:**
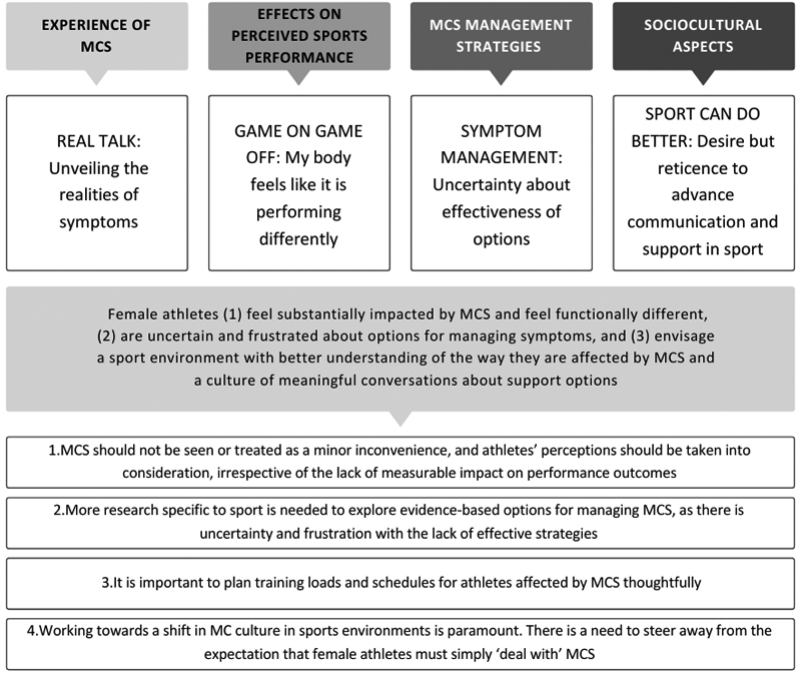
Summary diagram showing the overview of themes, conclusions and practical recommendations.

This qualitative exploration of female athletes' experiences with MCS, indicates that regardless of whether performance is measurably affected, female athletes feel substantially impacted by MCS and feel functionally different. There is uncertainty and frustration about options for managing symptoms. Female athletes in this study envisage a sport environment with better understanding of the way female athletes are affected by MCS and, despite reticence by some, a culture of meaningful conversations about support options is desired. Figure 1 presents a summary diagram of the findings and practical recommendations.

Participants in our study described their experiences with MCS as largely negative, irrespective of HC use, in line with findings from cross-sectional quantitative studies (5, 11) and recent qualitative studies (13, 15). These descriptions strongly demonstrate that some female athletes are negatively impacted by an array of MCS which have a wide-ranging influence on wellbeing and perceived performance. Our findings demonstrate an interplay between physical and mental symptoms that can exacerbate the impact of MCS. Historically, there has been a tendency to dismiss the impact of MCS or to “fix” symptoms with use of HCs. Although HCs are sometimes recognised as valuable tools for modifying and/or reducing the severity of some MCS, such as irregular and painful bleeding (3, 24), they do not completely eliminate negative MCS for many users (4). Medical literature now recognises a gender pain gap where women report difficulty having their pain legitimised (25) and are less likely to receive different treatment for pain than males (26). Consequently, socio-cultural normalisation of MC-related pain is increasingly being challenged (27). The widespread prevalence of MCS does not justify an expectation that female athletes will just “*deal with*” them. Findings from this study highlight that MCS are not minor inconveniences that are easily tolerated. They are impactful and, in many cases, distressing. MCS need to be given due consideration to support female athletes to speak up about what they are experiencing and continue to participate in sport and perform at their optimal level.

Research investigating whether the MC affects performance have typically demonstrated no measurable impact, as reviewed by Elliott-Sale et al. (10), and McNulty et al. (9) However, our findings indicate that athletes feel functionally different during the late luteal phase (i.e., high progesterone phase) and/or menstrual phase of their MC, equivalent to before and during withdrawal bleeding for HC users. Other studies using self-report questionnaires observed similar results, with most female athletes claiming menstruation as the most challenging phase of the MC (5, 6). Similarly in a recent survey study on 1086 soccer, orienteering and handball female athletes, participants perceived that their MC and HC cycles negatively affected their training and performance (11). Despite recognising that the quality of their performance was reduced, participants in our study typically felt it is necessary to “*train on*” regardless of how they felt. This mirrors findings from other studies, where athletes perceive MCS as an invalid reason to skip training and feel pressure to conceal menstrual issues (13, 28). Greater thought to options for modifying training in consideration of MCS is warranted. Our study documents the experiences of women who are continuing to train and compete. We have not captured the experience of women who have left sport. Therefore, attention should be given to how to best plan training loads and schedules when athletes are affected by MCS and whether better awareness and management would help retain females in sport.

Participants were frustrated by the lack of effective options to manage their MCS. Most participants used HCs for targeting specific symptoms like heavy bleeding, bleeding irregularities, skin blemishes, and painful bleeding. HCs are commonly prescribed for long-term relief of menstrual-related issues in both sports and the general population (4, 6, 29). However, in this study, some participants experienced unwanted side effects causing them to discontinue or switch HCs. The inconsistent effectiveness of HCs frequently left athletes in our study feeling frustrated. Similar results have been reported elsewhere (30). Nonsteroidal anti-inflammatory drugs (NSAIDs) and acetaminophen are often used for pain-related MCS relief, yet their long term use can cause negative side effects on other body systems like cardiovascular and renal (29). Furthermore, their potential negative side effects could extend to impact training adaptations (31). Participants in our study reported taking these medications largely for menstrual cramps, but their use was infrequent, varied in effectiveness, and there was a reluctance to rely on medication. Participants' reluctance was due to awareness of potential side effects and a preference for non-pharmacological pain relief methods. As a result, neither HCs nor pain relief medication appear to be universal MCS management strategies for participants in this study.

Athletes are generally encouraged to track their MC and HC cycles using accessible, safe, and efficient methods (32). Women who use active coping strategies, such as tracking for training and competition planning, may have an advantage over those who conceal or avoid their symptoms. A 2016 study on 217 non-athlete women (average 20 y/o, 59% oral contraceptive users) showed improvements in MCS-related discomfort, stress, and quality of life through active coping (33). Some athletes in our study implemented MCS tracking to understand their cycles and adjust training. However, others found tracking uninformative, inconvenient and stressful. Personal preference and potential negative psychological effects of MCS tracking should be considered. The unpredictable nature of MCS (i.e., changes from cycle to cycle) and daily changes [i.e., from “*feeling like a million bucks*' (P28) to “*feeling gross*' (P1)] experienced by participants add complexity, making it difficult for athletes to understand patterns and find support. This led to participants' hesitation in sharing MC information and a view that sharing was unnecessary unless a significant benefit was gained. The cost-benefit of MCS tracking requires further investigation.

Athletes in this study desired better awareness, understanding and communication about MCS yet there was reticence to talk about their MCS. This reticence was due to both embarrassment and a “*tough it out*” mentality to avoid being seen as “*weak*” or “*difficult*”. This dichotomy is common for women facing MCS, menstrual dysfunctions, and chronic pelvic and genital pain conditions, partly due to the fear of how new information will be received by those who do not experience the same issues (34). Previous negative experiences also affected some participants' confidence and openness to engage in MC conversations, making them less likely to share MC-related information. Similar findings have been highlighted by other qualitative studies in female athletes (13, 15, 35). Participants' discomfort discussing menstruation can be understood through the concept of stigma (36), which highlights social exclusion, devaluation, or internal shame when someone's attributes deviate from societal norm, as it is the case of menstruation in a social context (37). This may lead athletes to conceal or downplay menstrual-related experiences, as these could lead them to be perceived as weak or out of the ordinary. Athletes in our study challenged coaches, especially male coaches, to take initiative for improving communication about MCS. However, the conflict between desire and reticence to progress communication about MCS indicates that a broader cultural shift is required to progress sport to a place where meaningful communication about MCS can occur.

## 5 Practical recommendations

These findings offer several practical opportunities for improvement to support female athletes with MCS, helping them continue participating in sport and perform at their best:

1. MCS should not be seen or treated as a minor inconvenience, and athletes' perceptions should be taken into consideration, irrespective of the lack of measurable impact on performance outcomes.
2. More research specific to sport is needed to explore evidence-based options for managing MCS, as there is uncertainty and frustration with the lack of effective strategies.
3. It is important to plan training loads and schedules for athletes affected by MCS thoughtfully.
4. Working towards a shift in MC culture in sports environments is paramount. There is a need to steer away from the expectation that female athletes must simply “*deal with*” MCS.

## 6 Limitations

Recruitment targeted women who had experienced at least one negative MCS for at least the past six MCs or HC cycles hence our data captures the views and experiences of females affected by MCS rather than a broader sample. Our sample includes a limited range of ethnicities, and did not include athletes below 19 or above 32 y/o. This means we missed perspectives from athletes in other cultures, adolescent athletes, and older athletes. We interviewed women who are continuing to train and compete. It would be interesting for future studies to capture the experiences of women who have left sports due to their MCS.

## 7 Conclusion

This study underscores the significant impact of MCS on female athletes' perceived wellbeing, performance and sports engagement. Despite individual variations in MCS experiences, athletes consistently reported negative impacts, frustration with available management options, and dissatisfaction with the sports culture around MCS, regardless of their sports background or use of HCs. Results revealed a pressing need for a more informed and supportive approach to MCS within sports, calling for more research on evidence-based options for managing MCS and a cultural shift in sports environments. However, a notable reticence to sharing MC and HC related information was stressed, hindering communication and progress in this space. Addressing barriers related to insufficient MC knowledge and moving away from the conventional MC stigma and the long-standing expectation that female athletes should endure MCS, will promote openness and constructive MC-related discussions. Overall, these results underscore the importance of developing athlete-centred training and support strategies, aiming to improve the overall experience, performance and wellbeing of female athletes in various sports.

## Data Availability

The raw data supporting the conclusions of this article will be made available by the authors, without undue reservation.
