# Supplementary material for: Sport can do better: female athletes' perspectives on managing menstrual and hormonal contraceptive cycle-related symptoms
Source: Front Sports Act Living. 2025 May 26;7:1597469. doi: 10.3389/fspor.2025.1597469 (PMC12146347; doi:10.3389/fspor.2025.1597469)
Supplement: Supplementary file 1 [file Table1.docx]

# Supplement

*Supplement 1.* Interview guide consisting of three parts: (1) introductory questions: demographic and sports-related; (2) main questions: Menstrual cycle (MC)/ Hormonal contraceptive (HC) status, menstrual cycle-related symptoms (MCS), MCS management strategies; (3) menstrual literacy knowledge acquisition, perception on ease of menstrual-related communication, and general questions from participants.

| Disclosure statement: |
| --- |
| Before we start, I would like to make you aware that our conversation will be recorded and kept confidential. Recording it would help me consider everything we discuss and refer to some points, as I will struggle to remember everything we talk about today.  However, you can stop this recording or conversation at any point if you wish, and there is no pressure to answer every question, if you don’t want to answer that is completely fine.  There also no right or wrong answers. What is important here are your own personal experiences and views relating to the menstrual cycle **OR** **hormonal contraceptive use.**  In this conversation we will talk through how you feel about menstrual cycle-related symptoms, any impact you might recall in relation to your daily life, training and competition, and any management strategies that you might use or have used. |
| Section 1: *Introductory Questions* |
| - What is your age? How do you describe your gender identity, and what pronouns do you use (he/she/other)? - Tell me a bit about your sport and the training you do. - At what level do you compete in this sport? - Have you always played and competed in that sport? |
| Section 2: *Main Questions* |
| - Tell me about your menstrual cycle, **OR hormonal contraceptive (HC) cycle**, do you or have you ever tracked it? - Could you share the methods or tools you typically use for tracking it [menstrual cycle]? - Do you use these methods also to track any menstrual symptoms? - Tell me more about what is, or not, captured by the tool. - Naturally cycling (NC) only: Thinking about your menstrual cycle, can you tell me what is your experience of bleeding [menstruation] is like? What generally happens leading up to and during your bleeding? - **HC users only: You have shared that you take hormonal contraceptives, can you tell me how and why you started hormonal contraceptives? What has your experience been like using them? Can you tell me what is your experience with your withdrawal bleeding? What generally happens leading up to and during a withdrawal bleed?** - Can you describe to me more what it feels like and how does it affect you? – physically, mentally, emotionally – - Do you do anything about it? If so, what? - You shared that you experience menstrual symptoms and/or discomfort, what sort of symptoms and/or discomfort you experience? - How do these affect you? - What is that like for you? - Can you think about how this might be affecting you in any other ways? - Think about the day where you have your heaviest bleeding. Does it impact you differently compared to any other day? - How would you describe the consistency of your symptoms from cycle to cycle? Do they tend to stay the same or do they vary significantly? - Can you tell me about whether you feel your menstrual cycle, **OR HC use**, impacts your training? What about tournaments/competitions/races? - Have you ever missed sessions due to your menstrual cycle, **OR any side effect of HC use**? Can you tell me about those circumstances? - ***Can you tell me more about this, why you feel that you can't miss training? - Do you usually adjust sessions due to your menstrual cycle, **OR any side effect of HC use**? OR would you like anything to be different? Can you tell me about those circumstances? - Can you tell me more about this, take me through how you adjust them OR why do you feel you would like [that] to be different? - Do you feel the quality of your training is any different? Do you feel you are putting less effort in during? And how do you feel after? - How do you feel that your concentration and cognitive function changes through your cycle? - Are there any things that you have tried or do try to prevent or manage your menstrual symptoms? - Can you talk me through the things you have tried? - Tell me more about this. How do you feel about [strategies used]? - How would you describe the effectiveness of these strategies based on your personal experience? - Why? How was life different? - Can you talk me through what you like and/or dislike about the things you have tried to manage your menstrual symptoms? - Is there anything diet wise that you feel could make a difference to your symptoms, and why is that? OR Do you have any thoughts about how what you eat might affect your menstrual symptoms, in either a positive or negative way? - Does the food you usually eat or crave change around your cycle? And where specifically in your cycle? - What about your appetite? Have you noticed any changes throughout your menstrual cycle? - How do you manage these changes? If you do |
| Section 3: *Menstrual Cycle Knowledge, Communication and Participant Questions* |
| - What are the most useful sources of information that you use to help you with your menstrual symptoms? - How comfortable do you feel talking to coaches and other team members about how your menstrual symptoms are affecting you? - How do you recall feeling about sharing menstrual cycle and HC use information with your peers and coaches? - Think back about previous years (early teens), what would you have liked your younger self to know? OR is there anything you would have liked to do differently? - Are there any things you think athletes/coaches need to know more about with respect to menstrual symptoms? - Is there anything you are unsure about or would like to understand better? - Is there anything else you would like to add about your experience and view of menstrual symptoms? |

*Supplement 2.* Consolidated Criteria for Reporting Qualitative Studies (COREQ) 32-item checklist.(23)

| No. Item | Guide questions/description | Section/Page Number |
| --- | --- | --- |
| Domain 1: Research team and reflexivity | | |
| Personal Characteristics | | |
| 1. Interviewer/facilitator | Which author/s conducted the interview or focus group? | Methods/Page 4 |
| 2. Credentials | What were the researcher’s credentials? E.g. PhD, MD | Methods/Page 3-4 |
| 3. Occupation | What was their occupation at the time of the study? | Methods/Page 3-4 |
| 4. Gender | Was the researcher male or female? | Methods/Page 3-4 |
| 5. Experience and training | What experience or training did the researcher(s) have? | Methods/Page 3-4 |
| Relationship with participants | | |
| 6. Relationship establishment | Was a relationship established prior to study commencement? | Methods/Page 4 |
| 7. Participant knowledge of the interviewer | What did the participants know about the researcher? e.g. personal goals, reasons for doing the research | Methods/Page 4 |
| 8. Interviewer characteristics | What characteristics were reported about the inter viewer/facilitator? e.g. Bias, assumptions, reasons and interests in the research topic | Methods/Page 3-4 |
| Domain 2: Study design | | |
| Theoretical framework | | |
| 9. Methodological orientations and Theory | What methodological orientation was stated to underpin the study? e.g. grounded theory, discourse analysis, ethnography, phenomenology, content analysis | Methods/Page 3 |
| Participant selection | | |
| 10. Sampling | How were participants selected? e.g. purposive, convenience, consecutive, snowball | Methods/ Page 4 |
| 11. Method of approach | How were participants approached? e.g. face-to-face, telephone, mail, email | Methods/Page 4 |
| 12. Sample size | How many participants were in the study? | Results/Page 5 |
| 13. Non-participation | How many people refused to participate or dropped out? Reasons? | Results/Page 5 |
| Setting | | |
| 14. Setting of data collection | Where was the data collected? e.g. home, clinic, workplace | Methods/Page 4 |
| 15. Presence of non-participants | Was anyone else present besides the participants and researchers? | Methods/Page 4 |
| 16. Description of sample | What are the important characteristics of the sample? e.g. demographic data, date | Results/Page 6 |
| Data collection | | |
| 17. Interview guide | Were questions, prompts, guides provided by the authors? Was it pilot tested? | Methods/Page 4 and Supplement/Page 24-25 |
| 18. Repeat interviews | Were repeat inter views carried out? If yes, how many? | Results/Page 5 |
| 19. Audio/visual recording | Did the research use audio or visual recording to collect the data? | Methods/Page 4 |
| 20. Field notes | Were field notes made during and/or after the interview or focus group? | Method/Page 4-5 |
| 21. Duration | What was the duration of the interviews or focus group? | Results/Page 6 |
| 22. Data saturation | Was data saturation discussed? | Methods/Page 5 |
| 23. Transcripts returned | Were transcripts returned to participants for comment and/or correction? | Results/Page 5 |
| Domain 3: Analysis and findings | | |
| Data analysis | | |
| 24. Number of data coders | How many data coders coded the data? | Methods/Page 5 |
| 25. Description of the coding tree | Did authors provide a description of the coding tree? | Methods/Page 5 |
| 26. Derivation of the themes | Were themes identified in advance or derived from the data? | Methods/Page 5 |
| 27. Software | What software, if applicable, was used to manage the data? | Methods/Page 5 |
| 28. Participant checking | Did participants provide feedback on the findings? | Method/Page 5 |
| Reporting | | |
| 29. Quotations presented | Were participant quotations presented to illustrate the themes/ findings? Was each quotation identified? E.g. participant number | Results/Page 6-14 and Supplement/Page 25-27 |
| 30. Date and findings consistent | Was there consistency between the data presented and the findings? | Discussion/Page 14-16 |
| 31. Clarity of major themes | Were major themes clearly presented in the findings? | Results/Page 6-14 |
| 32. Clarity of minor themes | Is there a description of diverse cases or discussion of minor themes? | Results/Page -7 and Discussion/Page 14-16 |

*Supplement 3.* Symptomology summary and quote illustrations.

| ***Menstrual cycle-related symptom category*** | ***Menstrual cycle-related symptoms*** | ***Quotes*** |
| --- | --- | --- |
| Physical pain | Cramps  Lower back pain  Joint pain or stiffness  Headaches, migraines  Breast tenderness  Bodily discomfort  Mid-cycle abdominal pain  Mid-cycle headaches | *"So it’s [cramps] like in the lower part of your stomach, like in the bottom and... it’s like kind of enraged, like all comes in really close, like two closed fists and it just like makes you feel sick and it makes me feel like I can’t eat... like the only way to make you feel better is if you like folded in half”* (P14)  *“I notice it [hip and knee pain] a lot, as it is more difficult to move. Especially before, two or 3 days before I’m going to bleed, I notice it”* (P26)  *“The most uncomfortable for me is swollen breasts because it’s very painful and also, you know, when wearing a sports bra and running and stuff, that’s very, very painful. So I dread it”* (P18) |
| Sleep | Sleep disturbances  Mid-cycle sleep disturbance | *“My sleep [before and during bleeding], I noticed that it’s a bit more difficult to fall asleep and I might wake up during the night, or I might wake up a bit earlier, like my sleep feels a bit affected”* (P21)  *“In the middle of my cycle around ovulation time, I’d have one or two nights where I can’t sleep. I wake up and then I can’t go back to sleep. I also have headaches for one or two days”* (P19) |
| Energy levels | Low energy  Exhaustion  Heavy body  Lethargy | *“I get really frustrated and I get really fatigued really quickly… even just sitting and typing… sucks the life out of me… Usually I feel frustrated because I know that I'm not operating at full capacity…”* (P25)  *“In the third week of my cycle, I have very heavy legs. If I go to training, I feel like I have no recovery at all. My legs feel sore without even doing anything”* (P19)  *"Your energy is limited and you have to think about like where am I putting the energy available?... it's just constantly tired and feeling like everything takes double or triple the effort that it normally would”* (P27) |
| Gastrointestinal disturbances | Constipation  Diarrhea  Nausea and vomiting  Bloating  Gassiness  Water retention | *"Maybe sometimes, like some digestive problems... It’s more like constipation, but then on the beginning of the period, I have sometimes diarrhea, not fun”* (P18)  *“… So if it’s [cramping pain] really bad, also I am feeling sick and I have to throw up”* (P17)  *"Flatulence is a big one which will come up around that time [during bleeding], even leading up to it... So it's just like everything's churning away in there”* (P11)  *“Usually I start to retain quite a lot of liquids [before bleeding], so I feel a bit more bloated”* (P21) |
| Bleeding | Heavy bleeding  Mid-cycle spotting | *“I have previously filled up super menstrual cups in a matter of hours and I have bled through overnight period underwear in the last six months”* (P25)  *“I do spot every month or almost every month… It’s about just before the fertile window* *So like maybe four days after the last day of my period”* (P15) |
| Self-perception | Self-critical  Negativity  Self-doubt  Feeling let down  Frustration | *"I just don't cope with feedback very well or I'm quite self-critical like there's a lot of negative self-hatred that's happening in that week [premenstrual] unfortunately. So lots of criticism, bit of anxiety, but mostly just tears. That happens every month”* (P1)  *“It’s maybe been affecting my perception of self. I’ve been just a bit harder on myself because of that, which is not something you wanna be doing because that can, longer* *down the track, affect your self-esteem and confidence and lead into other areas of your life”* (P12)  *“If I had to go for a run, I would just think well, there’s no way I’m gonna be able to run as far as I planned to… it’s almost like I’m just kind of setting myself up to fail”* (P24)  *“It’s annoying because you feel that you need to be at a certain point in your performance, in your hype and you know, everything. So you sort of expect something from yourself, which maybe you’re not giving”* (P21) |
| Emotions | Emotional  Mood swings  Urges to cry  Irritability  Depression  Increased anxiety/worry  Reactivity towards others  Increased sensitivity  Low resilience | *“I’m more emotional, so that’s something that stresses me… I start to cry more quickly, get annoyed more quickly”* (P9)  *“Once in a while, not every cycle, I would find myself maybe slightly more sensitive to things, a bit more emotionally sensitive to things that wouldn’t normally bother me, but might personally make me a bit sad”* (P13)  *"I'll become a bit more downtrodden and not quite depressed, but um yeah, I noticed a significant change in my mood. I'm a lot more temperamental and likely to start feeling sensitive about things”* (P11)  *“...It’s always really uncomfortable [being on her period] because you are worried really. Have you got a string hanging out or whatever”* (P14)  *"I just feel like I have a lower emotional resilience, if that makes sense. So something at another time in my cycle that wouldn't bother me or make me upset, would bother me and make me upset”* (P2) |
| Behaviour | Less social  Poor engagement | *“Just feeling super low energy. Don’t wanna go out and, you know, see friends. But you wanna be social, but you just don’t have the energy for it... You want to crawl into like your bed and just cover yourself with a blanket and be warm and sleep. But you know that that's not gonna be productive. And so it's very frustrating”* (P27)  *“… When you’re kind of like distracted and feeling glum, you don’t really feel like a chat like, you know … In terms of going out of my way to like, talk to people, I wouldn’t normally know, I was kind of like, I don’t really have the energy like to make small talk with people”* (P16)  *“I think that when I’m on my period, my like engagement levels are less. And then like when they [teammates] try to explain a joke to me, I’m just not even listening. Like I’m just completely in another world, like looking straight through them”* (P14) |
| Decision making and concentration | Overwhelmed  Poor judgement  Brain fogginess  Reduced mental focus/concentration  Reduced reaction time | *"I will find decisions a lot harder than I would normally… A decision that I might have done one week… will feel a lot harder…. You’re like, 'why can’t I make this decision? I did it last week. Now it feels like 100 times harder. What’s going on?'. You sort of get that mental spiral… my training’s going awful. Like I’m not keeping up”* (P8)  *“One of the symptoms I can get is just like feeling quite overwhelmed and just not being in the present because you’re busy thinking about everything [symptoms and bleeding wise]”* (P12)  *“My reaction times would be slower. I would be slower to make decisions... It was sort of manifesting in me being slower on the boat, more tense when I couldn’t afford to be for my own and my crew’s safety”* (P13) |
| Diet-related | Appetite changes  Cravings | *“I feel like I just can’t eat enough food… I almost need a whole another meal… it almost feels like I’m just like engorging myself with food… I feel like, Oh my God, like, I’m just so fat because, like, I’m just eating all this food”* (P24)  *“I feel like I'm like so much more hungry. I could eat like carbs just all day. Ohh, like chocolate. Like doughnuts, anything sweet and unhealthy”* (P3) |
| Other | Heat perception  Skin blemishes | *“With my training, I find that my body temperature just skyrockets and… I can be just like sweating like an absolute pig in a session and then a couple of days later I get my period and say, OK, that’s why”* (P8)  *“I get a little bit of acne… I usually get like a couple of pimples just before my period starts and then I get like some more pimples kind of around my jawline towards the end of my period”* (P24) |

*Supplement 4.* Individual participant sports background, Tier levels,(24) menstrual cycle (MC) characteristics, and cycle tracking preferences. OCP = Oral contraceptive pill; IUD = Intrauterine device (IUS, intrauterine system); BBT = Basal body temperature.

| **No** | **Age** | **Sport, Years in sport, Tier** | **Naturally cycling OR Hormonal contraceptive use** | **Cycle tracking and method** | **Records cycle-related symptoms** |  | **No** | **Age** | **Sport, Years in sport,**  **Tier** | **Naturally cycling OR Hormonal contraceptive use** | **Cycle tracking and method** | **Records cycle-related symptoms** |
| --- | --- | --- | --- | --- | --- | --- | --- | --- | --- | --- | --- | --- |
| 1 | 29 | Triathlon, 2 years, Tier 2 | Naturally cycling | Yes, Apple Health | Yes, regularly |  | 16 | 25 | Canoe polo, 10 years, Tier 3 | OCP (Estelle 35 ED) | No | No |
| 2 | 23 | Water polo, 6 years, Tier 4 | IUD Mirena ® | No | No |  | 17 | 30 | Ultimate frisbee, 2 years, Tier 2 | Naturally cycling | Yes, BBT | Yes, regularly |
| 3 | 27 | Triathlon, 4 years, Tier 4 | Naturally cycling | Yes, Ōura ring and Natural Cycles (BBT) | Yes, regularly |  | 18 | 25 | Ultimate frisbee, < 1 year, Tier 2 | Naturally cycling | Yes, Apple health | Yes, irregularly |
| 4 | 24 | Water polo, 15 years, Tier 4 | Naturally cycling | Yes, calendar based | Yes, irregularly |  | 19 | 26 | Soccer, 15 years, Tier 2 | Naturally cycling | Yes, FitRWoman app | Yes, regularly |
| 5 | 31 | Triathlon, 12 years, Tier 4 | Naturally cycling | Yes, Garmin connect | Yes, regularly |  | 20 | 28 | Water polo, 13 years, Tier 4 | OCP (LEVLEN® ED) | Yes, calendar based | No |
| 6 | 22 | Soccer, 7 years, Tier 3 | Naturally cycling | Yes, Apple health | No |  | 21 | 27 | Powerlifting, 6 years, Tier 3 | Naturally cycling | No | No |
| 7 | 32 | Running, 1 year, Tier 2 | OCP (Yasmin ®) | No | No |  | 22 | 28 | Olympic lifting, 2 years, Tier 2 | Naturally cycling | No | No |
| 8 | 25 | Triathlon, 2 years, Tier 2 | Naturally cycling | Yes, Apple health | Yes, regularly |  | 23 | 20 | Netball, 9 years, Tier 2 | Naturally cycling | Yes, Clue app | No |
| 9 | 28 | Ultimate frisbee, 10 years, Tier 2 | OCP (Femme-Tab ED 20/100) | No | No |  | 24 | 28 | Irish dancing/ 20 years/ Tier 3 | IUD Mirena ® | Yes, FitRWoman app | Yes, regularly |
| 10 | 26 | Pole sport, 4 years, Tier 3 | OCP (Femme-Tab ED 20/100) | No | No |  | 25 | 26 | Bodybuilding, 2 years, Tier 2 | IUD Mirena ® | Yes, Ōura ring | Yes, regularly |
| 11 | 24 | Powerlifting, 3 years, Tier 4 | OCP (ELEANOR 150/30 ED) | No | No |  | 26 | 23 | Rugby, 3 years, Tier 2 | Naturally cycling | Yes, Apple health | No |
| 12 | 22 | Squash, 13 years, Tier 4 | IUD Mirena ® | No | No |  | 27 | 26 | Ultimate frisbee, 5 years, Tier 2 | IUD Mirena ® | Yes, Apple health | Yes, regularly |
| 13 | 25 | Sailing, 13 years, Tier 4 | Naturally cycling | Yes, Clue app | No |  | 28 | 27 | Triathlon, 4 years, Tier 4 | Naturally cycling | Yes, Training Peaks | No |
| 14 | 19 | Hockey, 13 years, Tier 3 | Naturally cycling | Yes, Flow app | Yes, irregularly |  | 29 | 27 | Soccer, 20 years, Tier 2 | Naturally cycling | Yes, Flow app | Yes, irregularly |
| 15 | 27 | Ultimate frisbee, 3 years, Tier 2 | Naturally cycling | Yes, Apple health | Yes, regularly |  | 30 | 29 | Figure skating, 6 years, Tier 3 | IUD Mirena ® | Yes, Flow app | Yes, regularly |

23. Tong A, Sainsbury P, Craig J. Consolidated criteria for reporting qualitative research (COREQ): a 32-item checklist for interviews and focus groups. Int J Qual Health Care. 2007 Sep 16;19(6):349–57.

24. McKay AKA, Stellingwerff T, Smith ES, Martin DT, Mujika I, Goosey-Tolfrey VL, et al. Defining Training and Performance Caliber: A Participant Classification Framework. Int J Sports Physiol Perform. 2022 Feb 1;17(2):317–31.
